# FAMILY CARE PARTNER’S PREPAREDNESS IN CARING FOR HOSPITALIZED PERSONS WITH DEMENTIA

**DOI:** 10.1093/geroni/igae098.1363

**Published:** 2024-12-31

**Authors:** Anju Paudel

**Affiliations:** The Pennsylvania State University, University Park, Pennsylvania, United States

## Abstract

Many care partners are inadequately prepared to meet the care needs of hospitalized persons with dementia, and little is known about care partner characteristics associated with preparedness. This paper explores the care partner characteristics associated with preparedness in caring for hospitalized persons with dementia at discharge, 2 months, and 6 months post discharge. Following bivariate associations, we conducted stepwise regression in a sample of 461 care partners of hospitalized persons with dementia who participated in Fam-FFC. On average, care partners were 61.81 years old (SD = 14.19) and primarily female (n=334, 72.5%). Care partners reported having some preparedness in general and it continued to increase from discharge (23.75, SD=6.78) to 2 months (24.5, SD=6.49) and 6 months (26.35, SD=6.73). Multiple care partner characteristics were associated with preparedness at discharge [age (b= -0.071; p<.001), burden (b= -0.283; p<.001), and depression (b= -0.284; p<.01)], 2 months [burden (b= -0.226; p<.001), strain (b= -0.144; p<.05), depression (b= -0.185; p<.05)] and 6 months [burden (b= -0.164; p<.01), strain (b= -0.183; p<.05), depression (b= -0.279; p<.01)] post discharge. While care partners’ feelings of greater burden and depression were associated with lower preparedness at all time points, care partners’ higher age was associated with lower preparedness at discharge only and care partners’ feelings of higher strain was associated with lower preparedness at 2- and 6-months post discharge. Findings suggest the need to address care partners’ feelings of burden, strain, and depression to optimize their preparedness in meeting unique care needs of hospitalized persons with dementia.

